# Computerised Analysis of Telemonitored Respiratory Sounds for Predicting Acute Exacerbations of COPD

**DOI:** 10.3390/s151026978

**Published:** 2015-10-23

**Authors:** Miguel Angel Fernandez-Granero, Daniel Sanchez-Morillo, Antonio Leon-Jimenez

**Affiliations:** 1 Biomedical Engineering and Telemedicine Research Group, University of Cadiz. Avda. de la Universidad, 10, 11519 Puerto Real, Cadiz, Spain; E-Mail: daniel.morillo@uca.es; 2 Department of Automation, Electronics and Computer Architecture and Networks, University of Cadiz. Avda. de la Universidad, 10, 11519 Puerto Real, Cadiz, Spain; 3 Pulmonology, Allergy and Thoracic Surgery Unit, Puerta del Mar University Hospital, 11009 Cadiz, Spain; E-Mail: anleji@hotmail.es

**Keywords:** chronic obstructive pulmonary disease, COPD, data-driven, early detection, exacerbation, lung sounds, machine learning, PCA, prediction, respiratory sounds, sensor, support vector machine, SVM, symptoms, telehealth, telemonitoring

## Abstract

Chronic obstructive pulmonary disease (COPD) is one of the commonest causes of death in the world and poses a substantial burden on healthcare systems and patients’ quality of life. The largest component of the related healthcare costs is attributable to admissions due to acute exacerbation (AECOPD). The evidence that might support the effectiveness of the telemonitoring interventions in COPD is limited partially due to the lack of useful predictors for the early detection of AECOPD. Electronic stethoscopes and computerised analyses of respiratory sounds (CARS) techniques provide an opportunity for substantial improvement in the management of respiratory diseases. This exploratory study aimed to evaluate the feasibility of using: (a) a respiratory sensor embedded in a self-tailored housing for ageing users; (b) a telehealth framework; (c) CARS and (d) machine learning techniques for the remote early detection of the AECOPD. In a 6-month pilot study, 16 patients with COPD were equipped with a home base-station and a sensor to daily record their respiratory sounds. Principal component analysis (PCA) and a support vector machine (SVM) classifier was designed to predict AECOPD. 75.8% exacerbations were early detected with an average of 5 ± 1.9 days in advance at medical attention. The proposed method could provide support to patients, physicians and healthcare systems.

## 1. Introduction

Chronic obstructive pulmonary disease (COPD) is a primary cause of chronic morbidity and ranked as the third commonest cause of death in the world between 1990 and 2010 [1]. It has aroused a growing research interest as a major public health concern because of its mortality, prevalence and the resulting increased use of healthcare resources.

COPD is mainly caused by long-term exposure to noxious particles or gases and causes a progressive and persistent airflow limitation [2]. Assessment of COPD is based on clinical symptoms, future risk of exacerbations, identification of comorbidities and the severity of obstructive spirometry defined by the degree of airflow limitation evaluated by the forced expiratory volume in the first second (FEV_1_) [3]. Although COPD is currently under-diagnosed [4], overall prevalence in adults aged ≥40 years appears to lie between 9% and 10% [5] and all-cause mortality is increased in patients with this chronic condition.

Acute exacerbations of chronic obstructive pulmonary disease (AECOPD) are important episodes in the course of the disease associated with a significant increase in mortality, hospitalisation and health-care use and impaired quality of life. Exacerbations are defined as acute events, characterised by a worsening of the patient's respiratory symptoms from the stable state and beyond day-to-day variation, leading to a change in medical treatment and/or hospitalisation [6].

A recent cross-country study has shown that the greatest proportion of healthcare use is in primary care while hospital inpatient care accounts for greater percentage of total costs [7]. The largest component of total costs is attributable to admissions because of AECOPD [8].

The focus on reducing AECOPD may help reducing the costs linked to the disease management. Furthermore, it is well accepted that patients who early recognize AECOPD symptoms present a better health-related quality of life (HRQOL) and a lower risk of hospitalisation [9,10]. Therefore, early detection and prompt treatment of AECOPD might have important public health implications. It could help to improve HRQOL, to reduce the risk of hospitalisation and consequently the burden of the disease. The remote monitoring of patients with COPD for early detecting AECOPD is a primary objective in the research of chronic respiratory diseases since early treatment of exacerbations may be translated into improvements in outcomes. Recently, studies centered on early detection of AECOPD on a day-to-day basis through telehealth approaches have been reported. The works published have been supported on either physiological data [11,12], clinical diaries [13,14,15,16] or a combination of them [17,18,19,20].

In spite of the efforts made, the effectiveness of the interventions of home based remote monitoring in patients with COPD is unclear and further work is required [21]. A number of primary factors may be linked to the underlying causes of failure in the purpose of telemonitoring interventions applied to early detect and address AECOPD. Firstly, the lack of useful early predictors of an exacerbation is limiting the effectiveness of telemonitoring in COPD. Self-reporting of symptoms is subject to a perceptual bias (*i.e.*, difficulty of assessing symptoms) and does not allow capturing many of the features that are useful for clinical decision making. Furthermore, physiological parameters have not proved to be able to predict AECOPD, either because they change late in the time course of exacerbation or they cannot be measured reliably [22]. Secondly, poor compliance with reporting of signs and symptoms affects telemonitoring performance. Low compliance is mainly attributable to the use of devices that are not generally well accepted in real-world medical applications, especially in the case of elderly chronic patients with daily computerised tasks [23,24].

Accordingly, finding predictors with clinical reliability is a priority for the future design and development of interventions of home-based telemonitoring in COPD, as evidenced from the TELESCOT randomised controlled trial, a nested qualitative study about the impact of a telemetric COPD monitoring service [25].

Auscultation is a widely used tool in clinical practice for the detection of respiratory diseases. The availability of electronic stethoscopes and techniques of computerised analysis of respiratory sounds (CARS) are an opportunity for substantial improvement in the diagnosis of respiratory diseases like COPD.

The time course of AECOPD is characterised by a significant increase of airway obstruction and mucus production [26]. Abnormal respiratory sounds like wheezes and rhonchi are a manifestation of the latter conditions [27] and appear as key symptoms related to the pathophysiology of exacerbations of COPD. Consequently, changes in respiratory sounds are a clinical sign commonly reported during exacerbation episodes and have been analysed in scientific literature in different contexts [28,29,30]. In addition, the results of a very recent review suggest that adventitious respiratory sounds are mainly characterised by inspiratory and coarse crackles and expiratory wheezes in patients with COPD [31]. More recently, CARS have shown potential as a reliable marker in monitoring respiratory status in subjects with COPD [32]. Notwithstanding all these discoveries, unveiling or alteration of respiratory sounds in patients with COPD has been researched scarcely regardless of it being reported in about 35% of AECOPD [33].

In the absence of biomarkers or consolidated markers to early detect AECOPD, this study presents an exploratory study of the feasibility of using a respiratory sensor embedded in a special housing for self-use of ageing users, CARS and data-mining techniques for the remote early detection of the AECOPD according to the settings described in [23]. To our knowledge, there are no studies that have examined the evolution of respiratory sounds acquired by patients themselves as a part of the home-based telemonitoring intervention. This work hypothesizes that a computerised system can early detect changes in respiratory sounds during COPD exacerbations and that these changes can be classified thereby supporting in the prompt detection and treatment of AECOPD. The proposed mechanism by which individuals can easily and remotely record their respiratory sounds to enable early detection and prompt treatment of AECOPD could have important public health implications. The remainder of this paper is organised as follows: in Section 2, participants, methods and the infrastructure of the home telemonitoring system are described. Experimental results and performance using the proposed features and algorithms are presented in Section 3 and discussed in Section 4. Finally, conclusions and future work appear in Section 5.

## 2. Patients and Methods

### 2.1. Patients

In this observational pilot study, 16 COPD patients meeting the study inclusion criteria were identified and recruited in the Pulmonology, Allergy and Thoracic Surgery Department of the University Hospital Puerta del Mar of Cadiz (Spain) for the six-month pilot study. Inclusion criteria were patients aged over 60 years, with a smoking pack year history of greater than 20 pack years, a FEV_1_/forced vital capacity ratio of less than 70% post-bronchodilator in the stable phase of disease, with at least one hospital admission for exacerbation or two exacerbations treated with oral corticosteroids or antibiotics within last year, absence of cardiovascular comorbidities (heart failure or vascular disease), good mobile data coverage at home to effective and timely transmission of data to the remote management center and cognitive and motor capacities to handle a simple electronic device. The participants were equipped with a home base station and a sensor device to daily record respiratory sounds during 6 months at home. Before starting the pilot study, the patients were trained for the effective management of devices and procedures used in the experiment. 

### 2.2. Ethical Approval 

Ethical approval was obtained from the local Ethics Committee. Signed informed consent was obtained from all participants.

### 2.3. Sensor Device

Respiratory sounds are a clinical sign examined in the management of COPD. The authors of this study hypothesise that the remote monitoring of respiratory sounds could support the remote early detection of AECOPD. To this purpose, a microphone based device was designed. This sensor device was part of a multifunctional equipment able to acquire these data easily within a single user test [23].

An electret condenser microphone (ECM) was used. The performance of an ECM is defined by three parameters namely, (a) sensitivity, usually expressed in dBV/pa (dBV/10 μbar) and defined as the output voltage for a specified load condition and acoustic stimulus; (b) output impedance that represents the internal electric resistance as seen from output terminals and (c) frequency response (expressed in Hz) defined as the frequency range in which the microphone can receive sound. The selected microphone was a back electret condenser omnidirectional microphone. The technical specifications are summarised in Table 1 and follow the recommendations for the case of respiratory sounds acquisition [34].

**Figure 1:**
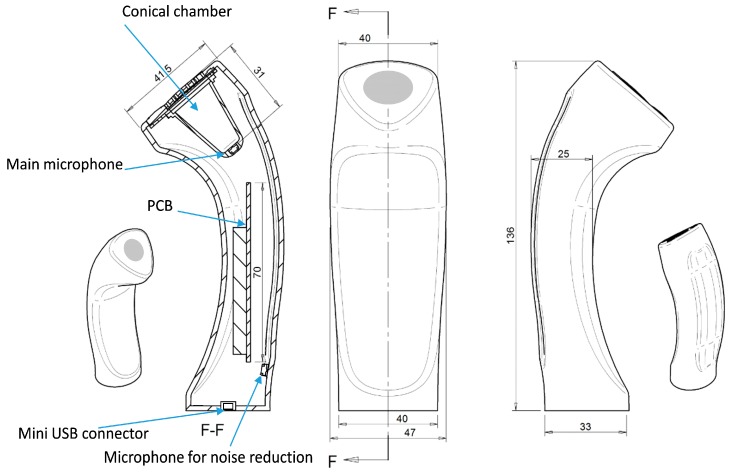
Remote respiratory sensor device. The mini USB connector, the printed circuit board (PCB) with the digitising hardware, the main microphone, the air-coupled conical chamber and the microphone for environmental noise reduction are shown.

The air chamber was conic shaped with a silicone diaphragm placed as part of the design for vented adherence to patient’s skin. A second microphone was used in order to record environmental noise. The device is basically depicted in Figure 1.

A microcontroller handled the analog to digital signal conversion with an analog to digital converter (ADC). ARM-based-32 bit microcontroller (MCU) with 12-bit analog-digital converter (ADC) was used. A USB port (high-speed mini-USB connection) was used to power the device and to transfer data to and from the sensor implementing a virtual serial port. Data from both microphones were received in the same data frame. Dominant frequency range of respiratory sounds is above 100 Hz and below 2000 Hz [35]. For an adequate estimate of frequency content, a sampling rate of 8000 Hz that safely satisfies the Nyquist criteria was used.

**Table 1:** Technical specifications of the selected microphone.

Frequency Band	50 Hz–10 kHz
Sensibility	44 dB
Impedance	2.2 K
Power	1–10 V
Current Consumption	500 μA (max)
S/N Ratio	55 dB (min)
External size	ϕ 3 mm × 1.5 mm
Mass	< 0.5 g
Operation Temperature	−30 °C to +70 °C
Omnidirectional	Yes

**Figure 2:**
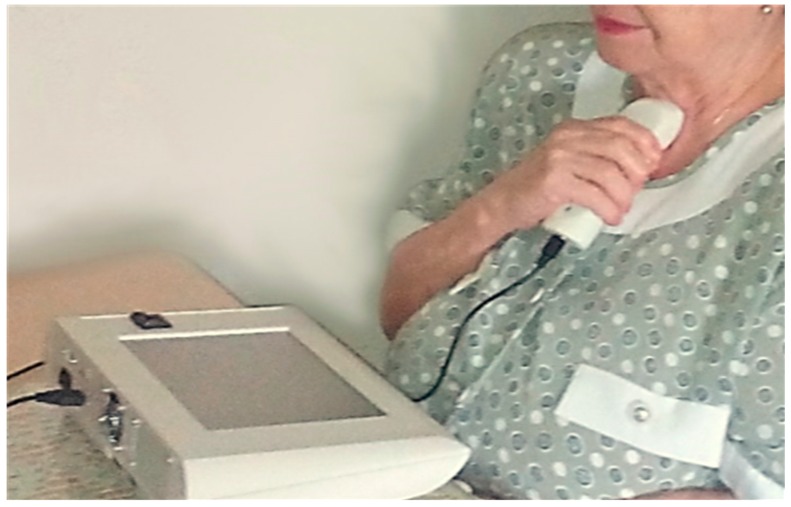
Photograph of a subject using the respiratory sensor at home during the process of respiratory sounds acquisition at the suprasternal notch. The base-station assistant steps the user through the process. The process is guided by the base station using visual and voice instructions for guidance.

Auscultation was performed on a daily basis by unsupervised patients at home. The sensor was placed on the suprasternal notch and the recording process was guided from the base-station by a multimodal interface. Details about the interface can be found in [23]. Figure 2 shows a photograph of a subject using the respiratory device at home during the process of respiratory sounds acquisition at the suprasternal notch. The base-station assistant stepped the user through the process using visual and voice instructions.

### 2.4. Pre-Processing and Feature Extraction

After removal of the DC components, an equi-ripple band-pass (BP) finite impulse response filter (FIR) from 100 to 2000 Hz with 80 dBs of attenuation out of the BP was applied. This filtering stage prevented aliasing and reduced the influence of heart, noise and muscle sounds. In order to enhance noise suppression, the output signal was then filtered with a recursive least squares (RLS) adaptive filter by using an estimated heart signal and the signal from the second environmental microphone [36].

After the pre-processing, the respiratory signal was converted into features that could be used for classification. Conventional methods of frequency analysis are not recommended because of the non-stationary dynamics of respiratory sounds. Instead, two different approaches were chosen. Firstly, windowing methods which apply overlapping windows and have an assumption of stationarity within a window were used including short time frequency transform (STFT), Mel-frequency cepstral coefficients (MFCC) and discrete wavelet transform (DWT). Secondly and in contrast, the Hilbert Huang transform (HHT) was used since HHT shows no limitations on window selection.

#### 2.4.1. Short-Time Fourier Transform

Each filtered respiratory sound signal was transformed into frequency domain by using STFT. This transformed signal was composed of a number of 25% overlapping frames of 64 ms (Hamming window, 512 samples). Amplitude normalization was done for each frame by dividing amplitudes of the frequency components for an STFT frame by the frame energy. After frame amplitude normalization step, frequency normalization was performed. For each frame, thirteen time-dependent parameters were estimated to quantify the spectral structure in each temporal segment:

- Characteristic frequencies: mean frequency and median frequency
- Spectral parameter: spectral crest factor
- Entropies: Shannon, Rényi and Tsallis entropies
- Relative power (RP) factors in octave bands: (100–200 Hz), (200–400 Hz), (400–800 Hz) and (800–2000 Hz)
- High order frequency moments: second order moment, skewness and kurtosis

Subsequently, in order to obtain a single value per parameter and respiratory signal, and to achieve a straightforward interpretation of the results, average and standard deviation of each of the parameters were calculated along all frames [37,38,39]. Therefore, a resulting subset of 26 parameters features was obtained. This features subset have been previously used by the authors of this study in the analysis of respiratory sounds in COPD patients [29,30].

#### 2.4.2. Mel-Frequency Cepstral Coefficients

Mel-Frequency Cepstral Coefficients (MFCCs) are widely used in speech signal processing. Furthermore, some researchers have previously used MFCC for respiratory sound analysis [40]. To compute the MFCCs of the respiratory sounds, the signal was divided into a number of overlapped frames and fast Fourier transform was applied to obtain the spectrum of each frame. The spectrum was then decomposed into a number of subbands using a set of Mel-scale band-pass filters. The MFCCs were calculated by applying the discrete cosine transform (DCT) on the logarithm of the magnitude response of the Mel-scale band-pass filters [41]. The first thirteen MFCCs were selected and average and standard deviation of each of them were calculated [39,42]. Consequently, an additional subset of 26 features were extracted.

#### 2.4.3. Discrete Wavelet Transform

**Figure 3:**
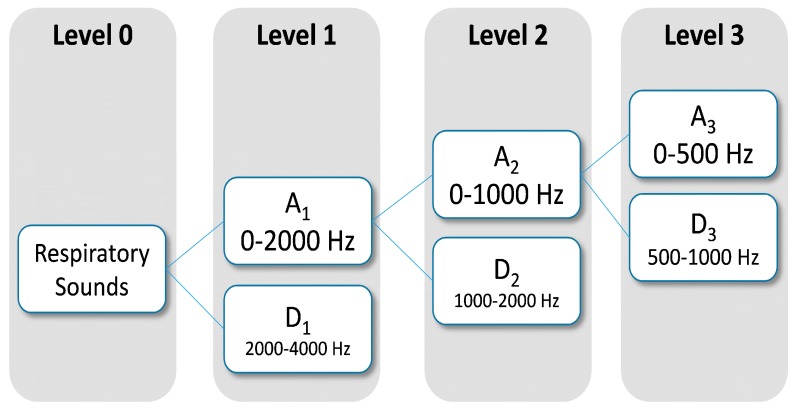
Subband decomposition of Discrete Wavelet Transform implementation. The used subband were A1, A2 and D2.

Dominant frequency components of the signal influence the number of levels of wavelets decomposition to be selected. Since frequency components of interest in respiratory sounds are mainly between 100 Hz and 2000 Hz, two levels were chosen for wavelet decomposition (Figure 3). The wavelets features were obtained from Daubechies 8 y Biorthogonal 1.5 [43,44] on the subbands, A_1_ (0–2000 Hz), A_2_ (0–1000 Hz) and D_2_ (1000–2000 Hz). From each subband six parameter were estimated: mean of the absolute values, average power, standard deviation, ratio of the absolute mean values of adjacent subbands, skewness and kurtosis. In total, 36 wavelets features were computed using DWT.

#### 2.4.4. Hilbert-Huang Transform

Hilbert-Huang Transform (HHT) is a method designed by Huang in 1998 applicable to nonlinear and non-stationary data [45]. The Empirical Model Decomposition (EMD) is the fundamental part of the Hilbert-Huang Transform algorithm. EMD allows decomposing, any complex time series dataset into a finite and usually small number of components described as intrinsic mode functions (IMF). Twelve instantaneous frequencies (IF) were obtained by applying the Hilbert transform to the IMFs [46,47]. Standard deviation and average of each IF were computed. Hence, 24 parameters were obtained using HHT.

#### 2.4.5. Dataset Creation

COPD prodromal phase is characterised by an increase of symptoms and may occur during seven days prior to the onset of an exacerbation [33]. Accordingly, the target was defined as a categorical dichotomous variable. Exacerbation onsets (accounted for self-administration of medication, unscheduled visits to emergency units and/or admissions) and the previous seven days were labelled with “1” and the rest of days were labelled with “0”. Periods of two weeks after the AECOPD, corresponding to recovery periods, were discarded [48]. The final dataset included for each row (*i.e.*, a monitored day for a patient) a total of 112 features and a dichotomous output that defined a warning state because of exacerbation. Features were smoothed by averaging over a sliding window of 14 days. Further details about this procedure can be found in reference [16].

#### 2.4.6. Principal Component Analysis and Model Selection

In order to reduce dimensionality of the problem, principal components analysis (PCA) was applied. PCA is an unsupervised analysis procedure that provides useful information about the relationship between measured variables. In PCA, the variance of most of the information is stored in the first few components. Therefore, dimensionality of the original set can be reduced with a minimal loss of information [49]. 

When PCA is used as a dimension reduction method for classification, cross-validation is often used to determine the number of factors in the model. In this study, PCA-SVM model selection algorithm with 10-fold cross validation was applied, where the data was divided into 10 subsets of equal size. In the *i-*th fold, the *i-*th subset was held out for validation and all other subsets were used as the training set to carry out PCA with Varimax rotation [50]. After PCA, a given number of principal components (PC) factors (which ranged from 1 to 112) was used to build the SVM classifier for each fold and the classification result of the validation subset was predicted by the PCA-SVM model. Then another subset was left out and subjected to the above procedures. This was repeated until all subsets had been left out once. In the end, all subsets had been classified once, and the number of correct classifications was logged.

The averaged geometric mean of sensitivity and specificity (G_M_) and the root mean squared error (RMSE) of the model for all folds was computed for each number of PCs. The number of PCs was decided as a trade-off between these two metrics. In addition, the average cumulative variance explained and eigenvalues were also estimated as a function of the number of components along cross-validation. This cross-validation was applied to identify the optimal number of principle components in terms of balance between G_M_ and RMSE and not to find the correct dimensionality of the PCA model.

### 2.5. SVM Prediction System

The reduced features obtained from PCA were used for training the classifier. In supervised learning, the model defines the effect inputs have on outputs. Outputs were assigned to exacerbation onsets. Therefore, prediction of exacerbations was faced as a classification problem and was addressed using a support vector machine (SVM) classifier. SVM are a supervised machine learning method used in both classification and regression and is closely related to classical neural networks. SVM achieves the classification separating optimally the data into two categories by constructing an N-dimensional hyperplane.

For classification, the input data are usually transformed to a high dimensional feature space where are linearly separable in comparison to the original space. Boser *et al.* [51] created a non-linear classifier that used a non-linear kernel function. The kernel used in this study was the radial basis function (RBF). This kernel is able to handle a non-linear relation between the attributes and the class labels [52].

The SVM classifier prediction performance is determined by parameters σ (the width of RBF kernel) and *C* (margin-losses trade-off). The best combination of σ and *C* was selected by a grid search. Search ranges were [0.001, 20] with a step equals 1 for σ [0.1, 50,000] with a step equals 10 for *C*. Each combination of parameter choices was checked using internal 4-fold cross-validation.

To reduce false-positive rate the classifier output was forwarded to a simple decision rule. An alarm because exacerbation was raised if and only if the SVM classifier generated a positive output for two consecutive days. This rule is an extrapolation of a clinical definition of symptom-based exacerbations [53] and allows partially screening bad isolated days from AECOPD [18].

**Figure 4:**
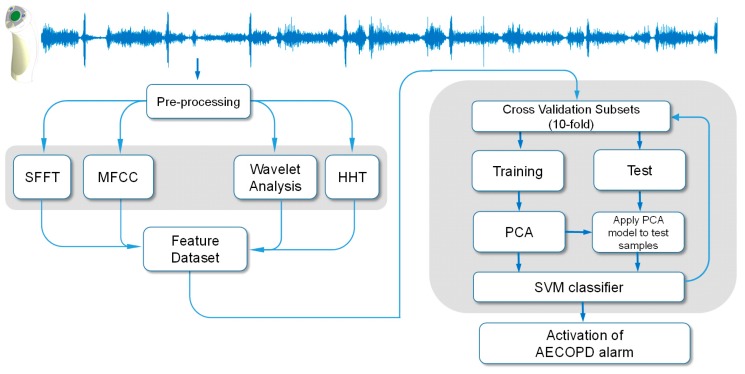
Flow diagram depicting the computational approach to the early detection of acute exacerbation of COPD.

Along the 10-fold cross-validation process, performance was assessed according to sensitivity, specificity, root mean squared error, geometric mean of sensitivity and specificity, accuracy, positive predictive value, negative predictive value and cumulative percentage of variance explained. MathWorks MATLAB^®^ (Natick, MA, USA) was used for statistical analysis and signal processing. A block diagram of the proposed respiratory sounds processing framework is illustrated in Figure 4.

## 3. Results

**Figure 5:**
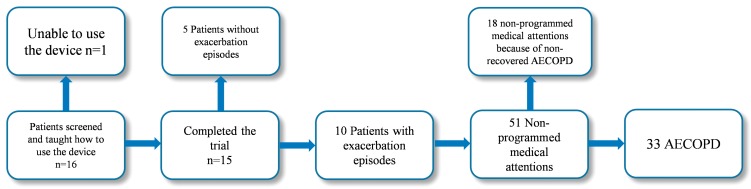
Flowchart with detailed information on patient participation, dropout and exacerbations along the pilot study.

The flowchart with detailed information throughout the study period on patient participation, dropout and exacerbations is shown in Figure 5.

Table 2 summarizes the demographic and clinical features of the participating subjects. Classification of COPD was based on the combined assessment of symptoms, airflow limitation and hospitalisations for exacerbations.

**Table 2:** Demographic and clinical characteristics of the participants that completed the trial (*N* = 15).

**Age (years)**	70.2 ± 6.6
Assessment of COPD* (%)	
** GOLD C (high risk, less symptoms)**	46.7% (7)
** GOLD D (high risk, more symptoms)**	53.3% (8)
**Confirmed AECOPD**	33
**Admissions**	10

Participants were 70.2 ± 6.6 years old and had a diagnosis of COPD confirmed by spirometry, classified in groups C and D according to GOLD guidelines [3].

An event-based definition of exacerbation was applied. An AECOPD was accounted for self-administration of medication, unscheduled visits to emergency units and/or admissions. Fifty-one interventions matched the applied definition of AECOPD. Eighteen out of the 51 medical attentions were associated with non-recovered exacerbations (*i.e.*, episodes took place during the recovery phase of the previous exacerbation and they were not considered for the study) and 33 of the events corresponded to AECOPD. Twenty-five out of the 33 selected events were due to non-programmed medical interventions, and the remaining 8 were accounted for self-management of medication.

**Figure 6:**
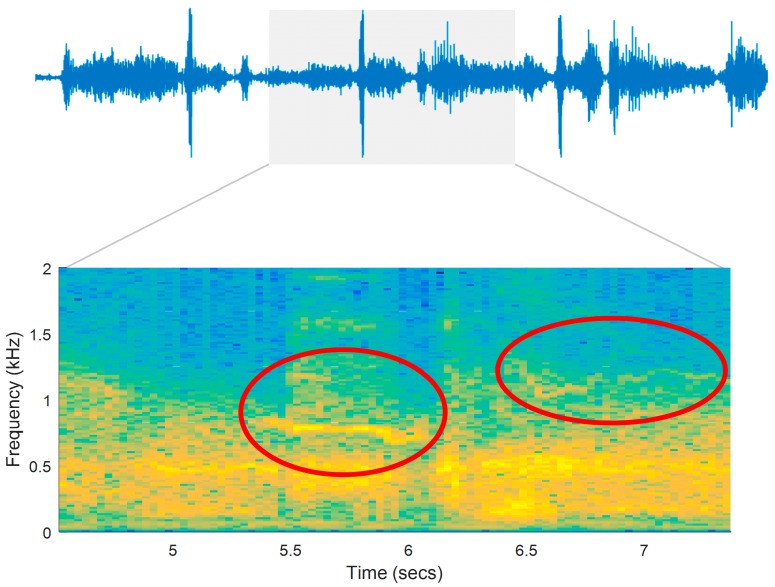
Interval of a respiratory signal and its spectrogram. The recording was remotely sent by a patient seven days prior to medical care (prodromal phase).

Figure 6 illustrates the respiratory sounds signal and its spectrogram for a patient in a register seven days prior to medical care (prodromal phase). Adventitious sounds (wheezes) are marked in the figure as possible indicators of airway obstruction.

**Table 3:** Classifier performance evaluation for the evaluated PCA-SVM model.

PCA Components	17
Cumulative variance explained	88.93%
Root mean squared error (RMSE)	0.2212
Geometric mean of sensitivity and specificity (G_M_)	0.849
True Positives (TP)	149 (10.64%)
True Negatives (TN)	1133 (80.87%)
False Positives (TP)	27 (1.93%)
False Negatives (FN)	53 (3.78%)
Sensitivity (Se)	73.76%
Specificity (Sp)	97.67%
Positive Predictive Value (PPV)	84.66%
Negative Predictive Value (NPV)	95.53%
False alarms of the system	3
AECOPD predicted	25 (75.8%)

**Figure 7:**
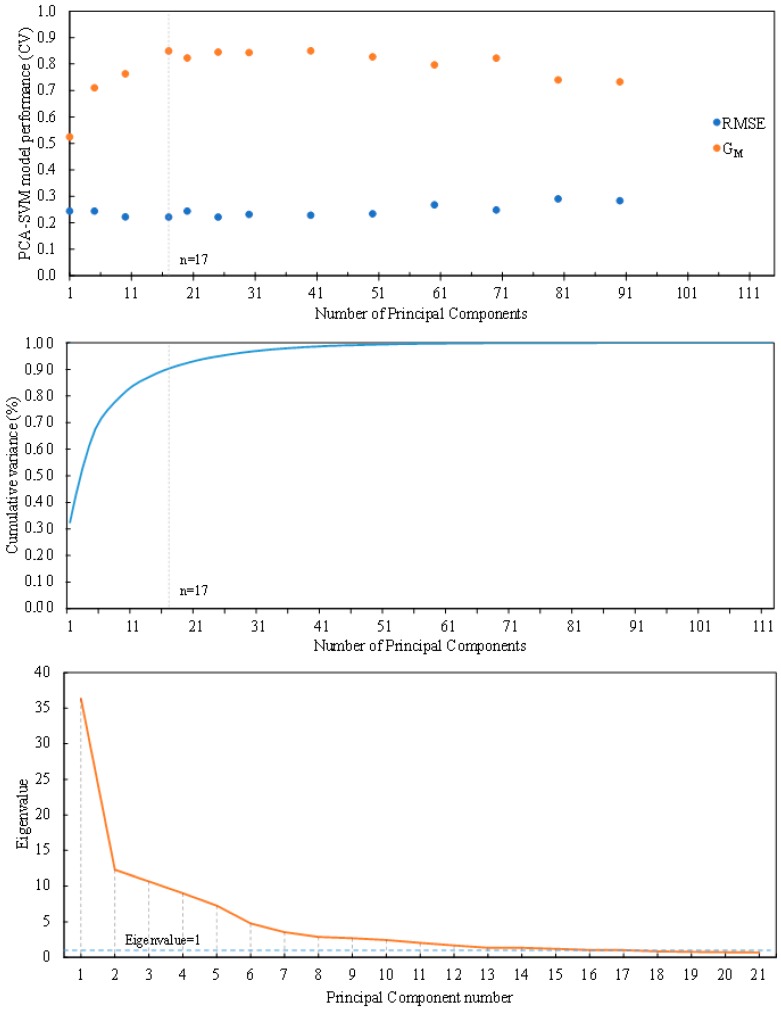
PCA-SVM model selection. Root mean squared error (RMSE) and geometric mean of sensitivity and specificity (G_M_) as a function of the number of principal components (**Top**). Cumulative proportion of variance explained *versus* factors (**Middle**). Cattell scree plot with factors on the x-axis and eigenvalues on the y-axis (**Bottom**).

PCA was used to reduce the dimensionality. 10-fold cross validation was used in the dataset to select the optimal PCA-SVM model. Model performance was evaluated for a number of PCs that ranged from 1 to 112 (Figure 7). A minimum in the RMSE, taken as a performance measure in this work, was achieved for 17 components. Best G_M_ values were achieved for 40 (0.850) and 17 (0.849) components. To corroborate the selection procedure, Cattell’s scree plot [54] and averaged along all cross-validation folds can be appreciated in Figure 7. Kaiser’s eigenvalue rule converged with seventeen components solution [55]. Seventeen principal components had eigenvalues greater than unity in 7 out of the 10 folds. These 17 components explained 88.93% of the total variance. As a consequence, a PCA model with 17 PCs was selected. The validated SVM classifier with 17 PCs provided a combination of sensitivity and specificity of 73.76% and 97.67% respectively, and a cumulative variance explained of 88.93% (Table 3). 

**Figure 8:**
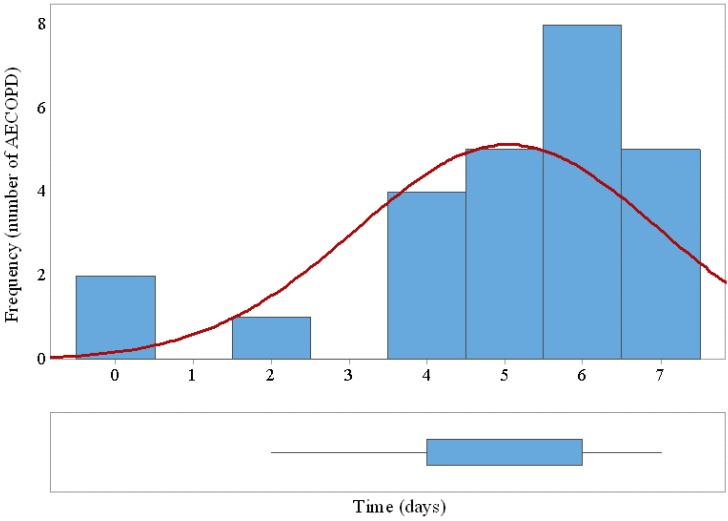
Histogram and box plot of AECOPD prediction margins based on performance of the SVM classifier and the decision rule. The horizontal axe indicates the days of early detection prior to medical attention.

The system was able to detect early 25 out of 33 (75.8%) AECOPD with a margin of 5 ± 1.9 days prior to the needed medical attention. Twenty-seven false positives generated at the output of the SVM classifier triggered only three false alarm states along the trial after applying the 2-days decision rule. Figure 8 shows the histogram and the box plot of prediction margins based on performance of the SVM classifier and the decision rule.

## 4. Discussion

This work presents, in the absence of consolidated biomarkers to predict AECOPD, an exploratory analysis of the feasibility of using a respiratory sensor embedded in a special housing tailored for self-use of ageing users, data-mining techniques and CARS for the remote prediction of the AECOPD. In a 6-month feasibility study, 16 patients with COPD were equipped at home with a sensor embedded in a self-tailored housing and a base-station to daily record their lung sounds. A PCA-SVM classifier and a decision rule were designed to early detect AECOPD. The proposed system has demonstrated a relevant predictive capacity, as 75.8% exacerbations were detected early an average of 5 ± 1.9 days in advance of medical attention.

To the authors’ knowledge a limited number of studies have been published on AECOPD prediction using only objective physiological measurements. In the study of Jensen *et al.* [11], SpO_2_, lung function, blood pressure, heart rate, weight and physical activity were used. The detection was treated as a pattern recognition problem, using linear discriminant functions. The system detected seven out of 10 exacerbations. In the study of Yañez *et al.* [56], a threshold-based algorithm that used home-based collected respiratory rate was assessed. Sensitivity of 66% and specificity of 93% were achieved in detecting episodes. Finally, in the study of Pedone *et al.* [57], SpO_2_, heart rate, weight and physical activity were measured at patient’s home. Based on an automatic threshold with a predefined range, only oxygen saturation could identify timely exacerbations, being able to cut by 33% the risk for hospitalizations.

One of the priorities for the development and future design of interventions of home-based telemonitoring in COPD is to find predictors with clinical reliability [21]. Recently, computerised analysis of respiratory sounds have shown potential as a reliable marker in monitoring respiratory status in subjects with COPD [32]. The detection of respiratory diseases in clinical practice is widely supported by auscultation. The availability of techniques of computerised analysis of respiratory sounds and electronic stethoscopes are an opportunity for important improvement in the diagnosis of COPD and other respiratory diseases. In spite of all these discoveries, unveiling or alteration of respiratory sounds in patients with COPD has been researched barely regardless it being reported in about 35% of AECOPD [33].

According to the achieved results, computerised analysis of collected home telehealth respiratory sounds appears to be a promising COPD monitoring tool. Telemonitoring of respiratory sounds might be used together with symptoms diaries to build a composite measure useful to predict AECOPD. Using the symptoms acquired by a multimodal diary solution, promising results have been obtained by the authors in previous works, with the same patients and during the same period of this study. A k-means classifier provided an overall accuracy of 84.7% in early detecting AECOPD. The system was able to predict AECOPD with a margin of 4.5 ± 2.1 days prior to the medical attention [16]. In addition, a probabilistic neural network (PNN) classifier was also designed and obtained a prediction accuracy of 80.5% for detection exacerbations defined according to symptoms criteria. Prediction margin was, as average, of 4.8 ± 1.8 days prior to onset [15].

There are some limitations to this study. The first issue is the small number of recruited patients due to the complexity in executing these kind of pilot studies. A second limitation is related to the six-month period selected for the trial. The more detrimental seasons for patients were included [58]. Therefore, the proposed system needs to be validated with a larger and less homogeneous patient population and during a larger period to improve statistical validity of cross-validation. It is expected that a larger sample size will improve the robustness and performance of the algorithm and will reduce the risk of over-fitting the predictive model, given that cross-validation was used for selection of PCA-SVM features on a small dataset. Finally, the proposed predictive model might be considered a “black box” since do not enable the clinical understanding of the mechanisms that rule the generation of the outcome.

This study is the first to explore the feasibility of using telemonitored respiratory sounds for early detection of acute exacerbation of COPD. Results achieved in this preliminary study showed that computerised analysis of respiratory sounds recorded at patients’ homes on a daily basis could be used as a complement of symptom diaries. The combination of respiratory sounds and symptoms diaries could improve the effectiveness of telehealth care systems for COPD management.

## 5. Conclusions

In summary, this preliminary study has aimed to illustrate the feasibility of developing a COPD exacerbation prediction model using only daily collected home telehealth respiratory sounds and a machine learning analytical approach. This work adds to the existing literature body being the first study to use unsupervised home telehealth respiratory sounds for the prediction of AECOPD.

Based on the findings of this study, the proposed system might be used to support the prediction of acute exacerbations of COPD on a day-to-day basis. It could be combined with symptoms diaries to build a composite measure that facilitates access to prompt treatment providing support to physicians, patients and healthcare systems. Next steps include the validation in separate and larger cohorts in order to improve patient specific adaptation, performance and robustness of the described method.

## References

[1] Patel J., Burney P.G.J., Newson R.B., Minelli C., Naghavi M. (2014). Global and regional trends in mortality from chronic obstructive pulmonary disease: Their relation to poverty, smoking and population change. Eur. Respir. J..

[2] Vestbo J., Hurd S.S., Agustí A.G., Jones P.W., Vogelmeier C., Anzueto A., Barnes P.J., Fabbri L.M., Martinez F.J., Nishimura M. (2013). Global strategy for the diagnosis, management, and prevention of chronic obstructive pulmonary disease: GOLD executive summary. Am. J. Respir. Crit. Care Med..

[3] Global Initiative for Chronic Obstructive Lung Disease (GOLD) Global Strategy for the Diagnosis, Management, and Prevention of Chronic Obstructive Pulmonary Disease. http://www.goldcopd.org/.

[4] Mannino D.M., Braman S. (2007). The epidemiology and economics of chronic obstructive pulmonary disease. Proc. Am. Thorac. Soc..

[5] Halbert R.J., Natoli J.L., Gano A., Badamgarav E., Buist A.S., Mannino D.M. (2006). Global burden of COPD: Systematic review and meta-analysis. Eur. Respir. J..

[6] Rodriguez-Roisin R. (2000). Toward a consensus definition for COPD exacerbations. Chest.

[7] Fletcher M.J., Upton J., Taylor-Fishwick J., Buist S.A., Jenkins C., Hutton J., Barnes N., van der Molen T., Walsh J.W., Jones P. (2011). COPD uncovered: An international survey on the impact of chronic obstructive pulmonary disease [COPD] on a working age population. BMC Public Health.

[8] Toy E.L., Gallagher K.F., Stanley E.L., Swensen A.R., Duh M.S. (2010). The economic impact of exacerbations of chronic obstructive pulmonary disease and exacerbation definition: A review. COPD.

[9] Wilkinson T., Wedzicha J.A. (2006). Strategies for improving outcomes of COPD exacerbations. Int. J. Chronic Obstr. Pulm. Dis..

[10] Wilkinson T.M.A., Donaldson G.C., Hurst J.R., Seemungal T.A.R., Wedzicha J.A. (2004). Early therapy improves outcomes of exacerbations of chronic obstructive pulmonary disease. Am. J. Respir. Crit. Care Med..

[11] Jensen M.H., Cichosz S.L., Dinesen B., Hejlesen O.K. (2012). Moving prediction of exacerbation in chronic obstructive pulmonary disease for patients in telecare. J. Telemed. Telecare.

[12] Mohktar M.S., Redmond S.J., Antoniades N.C., Rochford P.D., Pretto J.J., Basilakis J., Lovell N.H., McDonald C.F. (2015). Predicting the risk of exacerbation in patients with chronic obstructive pulmonary disease using home telehealth measurement data. Artif. Intell. Med..

[13] Walters E.H., Walters J., Wills K.E., Robinson A., Wood-Baker R. (2012). Clinical diaries in COPD: Compliance and utility in predicting acute exacerbations. Int. J. Chronic Obstr. Pulm. Dis..

[14] Mackay A.J., Donaldson G.C., Patel A.R.C., Singh R., Kowlessar B., Wedzicha J.A. (2014). Detection and severity grading of COPD exacerbations using the exacerbations of chronic pulmonary disease tool (EXACT). Eur. Respir. J..

[15] Fernández-Granero M.A., Sánchez-Morillo D., León-Jiménez A., Crespo L.F. (2014). Automatic prediction of chronic obstructive pulmonary disease exacerbations through home telemonitoring of symptoms. Biomed. Mater. Eng..

[16] Sanchez-Morillo D., Fernandez-Granero M.A., Jiménez A.L. (2015). Detecting COPD exacerbations early using daily telemonitoring of symptoms and k-means clustering: A pilot study. Med. Biol. Eng. Comput..

[17] Sund Z.M., Powell T., Greenwood R., Jarad N.A. (2009). Remote daily real-time monitoring in patients with COPD—A feasibility study using a novel device. Respir. Med..

[18] Burton C., Pinnock H., McKinstry B. (2015). Changes in telemonitored physiological variables and symptoms prior to exacerbations of chronic obstructive pulmonary disease. J. Telemed. Telecare.

[19] Van der Heijden M., Lijnse B., Lucas P.J.F., Heijdra Y.F., Schermer T.R.J. (2011). Managing COPD Exacerbations with Telemedicine.

[20] Hardinge M., Rutter H., Williams V., Toms C., Velardo C., Tarassenko L., Farmer A. (2014). Using a Mobile Health Application to Support Self-Management in COPD—Development of Alert Thresholds Derived from Variability in Self-Reported and Measured Clinical Variables. Am. J. Respir. Crit. Care Med..

[21] McKinstry B. (2013). The use of remote monitoring technologies in managing chronic obstructive pulmonary disease. QJM.

[22] Hurst J.R., Donaldson G.C., Quint J.K., Goldring J.J.P., Patel A.R.C., Wedzicha J.A. (2010). Domiciliary pulse-oximetry at exacerbation of chronic obstructive pulmonary disease: Prospective pilot study. BMC Pulm. Med..

[23] Sánchez-Morillo D., Crespo M., León A., Crespo Foix L.F. (2015). A novel multimodal tool for telemonitoring patients with COPD. Inform. Health Soc. Care.

[24] Sanders C., Rogers A., Bowen R., Bower P., Hirani S., Cartwright M., Fitzpatrick R., Knapp M., Barlow J., Hendy J. (2012). Exploring barriers to participation and adoption of telehealth and telecare within the Whole System Demonstrator trial: A qualitative study. BMC Health Serv. Res..

[25] Pinnock H., Hanley J., McCloughan L., Todd A., Krishan A., Lewis S., Stoddart A., van der Pol M., MacNee W., Sheikh A. (2013). Effectiveness of telemonitoring integrated into existing clinical services on hospital admission for exacerbation of chronic obstructive pulmonary disease: Researcher blind, multicentre, randomised controlled trial. BMJ.

[26] Wedzicha J.A., Hurst J.R. (2007). Structural and functional co-conspirators in chronic obstructive pulmonary disease exacerbations. Proc. Am. Thorac. Soc..

[27] Ceresa C.C., Johnston I.D.A. (2008). Auscultation in the diagnosis of respiratory disease in the 21st century. Postgrad. Med. J..

[28] Bergstresser T., Ofengeim D., Vyshedskiy A., Shane J., Murphy R. (2002). Sound transmission in the lung as a function of lung volume. J. Appl. Physiol..

[29] Sánchez Morillo D., Astorga Moreno S., Fernández Granero M.Á., León Jiménez A. (2013). Computerized analysis of respiratory sounds during COPD exacerbations. Comput. Biol. Med..

[30] Morillo D.S., Jiménez A.L., Moreno S.A. (2013). Computer-aided diagnosis of pneumonia in patients with chronic obstructive pulmonary disease. J. Am. Med. Inform. Assoc..

[31] Jácome C., Marques A. (2015). Computerized respiratory sounds in patients with COPD: A systematic review. COPD.

[32] Jacome C., Marques A. (2015). Computerized Respiratory Sounds Are a Reliable Marker in Subjects with COPD. Respir. Care.

[33] Seemungal T.A., Donaldson G.C., Bhowmik A., Jeffries D.J., Wedzicha J.A. (2000). Time course and recovery of exacerbations in patients with chronic obstructive pulmonary disease. Am. J. Respir. Crit. Care Med..

[34] Hadjileontiadis L.J. (2009). Lung Sounds: An Advanced Signal Processing Perspective.

[35] Palaniappan R., Sundaraj K., Ahamed N., Arjunan A., Sundaraj S. (2013). Computer-Based Respiratory Sound Analysis: A Systematic Review. IETE Tech. Rev..

[36] Gnitecki J., Hossain I., Pasterkamp H., Moussavi Z. (2005). Qualitative and quantitative evaluation of heart sound reduction from lung sound recordings. IEEE Trans. Biomed. Eng..

[37] Thongpanja S. (2013). Mean and median frequency of EMG signal to determine muscle force based on time-dependent power spectrum. Elektron. Elektrotech..

[38] Tucker S., Brown G.J. (2005). Classification of Transient Sonar Sounds Using Perceptually Motivated Features. IEEE J. Ocean. Eng..

[39] Eronen A. Comparison of features for musical instrument recognition. Proceedings of the 2001 IEEE Workshop on the Applications of Signal Processing to Audio and Acoustics (Cat. No.01TH8575).

[40] Palaniappan R., Sundaraj K., Sundaraj S. (2014). A comparative study of the svm and k-nn machine learning algorithms for the diagnosis of respiratory pathologies using pulmonary acoustic signals. BMC Bioinform..

[41] Lee C.-H., Shih J.-L., Yu K.-M., Lin H.-S., Wei M.-H. Fusion of Static and Transitional Information of Cepstral and Spectral Features for Music Genre Classification. Proceedings of the Asia-Pacific Services Computing Conference.

[42] Kizrak M.A., Bayram K.S., Bolat B. Classification of Classic Turkish Music Makams. Proceedings of the 2014 IEEE International Symposium on Innovations in Intelligent Systems and Applications (INISTA).

[43] Hashemi A., Arabalibiek H., Agin K. (2011). Classification of wheeze sounds using wavelets and neural networks. Int. Conf. Biomed. Eng. Technol..

[44] Kandaswamy A., Kumar C.S., Ramanathan R.P., Jayaraman S., Malmurugan N. (2004). Neural classification of lung sounds using wavelet coefficients. Comput. Biol. Med..

[45] Huang N.E., Shen Z., Long S., Wu M., Shih H., Zheng Q., Yen N.-C., Tung C., Liu H. (1998). The empirical mode decomposition and the Hilbert spectrum for nonlinear and non-stationary time series analysis. Proc. R. Soc. Lond. Ser. A Math. Phys. Eng. Sci..

[46] Içer S., Gengeç Ş. (2014). Classification and analysis of non-stationary characteristics of crackle and rhonchus lung adventitious sounds. Digit. Signal Process. A Rev. J..

[47] Xie G., Guo Y., Tong S., Ma L. (2014). Calculate excess mortality during heatwaves using Hilbert-Huang transform algorithm. BMC Med. Res. Methodol..

[48] Wedzicha J.A., Donaldson G.C. (2003). Exacerbations of Chronic Obstructive Pulmonary Disease. Respir. Care.

[49] Tsai F.S. (2010). Comparative Study of Dimensionality Reduction Techniques for Data Visualization. J. Artif. Intell..

[50] Jolliffe I.T. (2013). Principal Component Analysis.

[51] Boser B.E., Guyon I.M., Vapnik V.N. A Training Algorithm for Optimal Margin Classifers. Proceedings of the 5th Annual ACM Workshop on COLT.

[52] David L., Olson D.D. (2008). Advanced Data Mining Techniques.

[53] Langsetmo L., Platt R.W., Ernst P., Bourbeau J. (2008). Underreporting exacerbation of chronic obstructive pulmonary disease in a longitudinal cohort. Am. J. Respir. Crit. Care Med..

[54] Cattell R. (1966). The scree test for the number of factors. Multivar. Behav. Res..

[55] Kaiser H. (1960). The application of electronic computers to factor analysis. Educ. Psychol. Meas..

[56] Yañez A.M., Guerrero D., Pérez de Alejo R., Garcia-Rio F., Alvarez-Sala J.L., Calle-Rubio M., Malo de Molina R., Valle Falcones M., Ussetti P., Sauleda J. (2012). Monitoring breathing rate at home allows early identification of COPD exacerbations. Chest.

[57] Pedone C., Chiurco D., Scarlata S., Incalzi R.A. (2013). Efficacy of multiparametric telemonitoring on respiratory outcomes in elderly people with COPD: A randomized controlled trial. BMC Health Serv. Res..

[58] Jenkins C.R., Celli B., Anderson J.A., Ferguson G.T., Jones P.W., Vestbo J., Yates J.C., Calverley P.M.A. (2012). Seasonality and determinants of moderate and severe COPD exacerbations in the TORCH study. Eur. Respir. J..

